# Calmodulin as a major calcium buffer shaping vesicular release and short-term synaptic plasticity: facilitation through buffer dislocation

**DOI:** 10.3389/fncel.2015.00239

**Published:** 2015-07-01

**Authors:** Yulia Timofeeva, Kirill E. Volynski

**Affiliations:** ^1^Department of Computer Science, University of WarwickCoventry, UK; ^2^Centre for Complexity Science, University of WarwickCoventry, UK; ^3^University College London Institute of Neurology, University College LondonLondon, UK

**Keywords:** synaptic transmission, synaptic vesicles, short-term plasticity, calcium channels, modeling biological systems

## Abstract

Action potential-dependent release of synaptic vesicles and short-term synaptic plasticity are dynamically regulated by the endogenous Ca^2+^ buffers that shape [Ca^2+^] profiles within a presynaptic bouton. Calmodulin is one of the most abundant presynaptic proteins and it binds Ca^2+^ faster than any other characterized endogenous neuronal Ca^2+^ buffer. Direct effects of calmodulin on fast presynaptic Ca^2+^ dynamics and vesicular release however have not been studied in detail. Using experimentally constrained three-dimensional diffusion modeling of Ca^2+^ influx–exocytosis coupling at small excitatory synapses we show that, at physiologically relevant concentrations, Ca^2+^ buffering by calmodulin plays a dominant role in inhibiting vesicular release and in modulating short-term synaptic plasticity. We also propose a novel and potentially powerful mechanism for short-term facilitation based on Ca^2+^-dependent dynamic dislocation of calmodulin molecules from the plasma membrane within the active zone.

## Introduction

Calmodulin (CaM) is a major neuronal protein that acts as a key mediator of multiple Ca^2+^-dependent intracellular signaling cascades in the brain. CaM regulates synaptic transmission and synaptic plasticity via Ca^2+^-dependent binding to its target proteins in both the pre- and the post-synaptic compartments. These include protein kinases, adenylyl cyclases, calcineurin, Munc13s, and voltage-gated Ca^2+^ channels (VGCCs) (Xia and Storm, 2005; Pang et al., 2010; Sun et al., 2010; Lipstein et al., 2013; Ben-Johny and Yue, 2014). It has been recently demonstrated that CaM binds Ca^2+^ ions with much faster kinetics than other major neuronal Ca^2+^ buffers such as calbindin-D28k (CB), parvalbumin, and calretinin (Faas et al., 2011). However, in comparison to the other buffers direct effects of CaM-dependent Ca^2+^ buffering on action potential (AP)-evoked presynaptic Ca^2+^ dynamics and vesicular release have not been systematically studied.

In this work we performed realistic, experimentally constrained model simulations of AP-evoked presynaptic Ca^2+^ dynamics and synaptic vesicle fusion in small excitatory synapses. We compared the relative contributions of Ca^2+^ buffering by CB and CaM to modulation of vesicular release probability and short-term synaptic plasticity. Our simulations demonstrate that, at physiologically relevant concentrations, fast Ca^2+^ binding to the N-lobe of CaM has a dominant effect in inhibiting AP-evoked vesicular release. We also show that the predicted effect of CaM Ca^2+^ buffering on short-term synaptic plasticity strongly depends on the location and mobility of CaM molecules.

Finally, we propose a novel mechanism for a dynamic regulation of presynaptic strength based on Ca^2+^-dependent interaction of CaM with membrane-associated proteins that contain the isoleucine–glutamine (IQ) binding motif (e.g., neuromodulin and VGCCs) (Alexander et al., 1988; Xia and Storm, 2005; Ben-Johny and Yue, 2014). Our simulations demonstrate that Ca^2+^-induced dislocation of CaM molecules from the plasma membrane could lead to a significant reduction of Ca^2+^ buffering capacity within the active zone (AZ). This in turn, leads to an increase of vesicular release probability during repeated APs. Thus, AP-evoked dislocation of CaM may provide a powerful mechanism for short-term facilitation of synaptic transmission.

## Materials and methods

### Modeling of presynaptic Ca^2+^ dynamics

Three-dimensional modeling of dynamic AP-evoked presynaptic Ca^2+^ influx, buffering, and diffusion, on a millisecond timescale, was performed in the Virtual Cell (VCell) simulation environment (http://vcell.org) using the fully implicit finite volume regular grid solver and a 10 nm mesh. In contrast to the simplified steady-state and/or non-stationary single compartment models that are normally used to approximate presynaptic Ca^2+^ dynamics on tens to hundreds of milliseconds timescale (Neher, 1998; Sabatini and Regehr, 1998; Scott and Rusakov, 2006; Ermolyuk et al., 2012), no assumptions regarding Ca^2+^ buffer binding and/or diffusional equilibration were made in the VCell model used here. VCell simulations using a 10 nm mesh produced solutions for presynaptic Ca^2+^ dynamics at vesicular release sensors similar to those obtained in our previous work with a 5 nm mesh (Ermolyuk et al., 2013). The use of the larger mesh substantially increased the computation speed and allowed us to simulate Ca^2+^ dynamics in the whole presynaptic bouton on the longer time scale.

**Figure 1:**
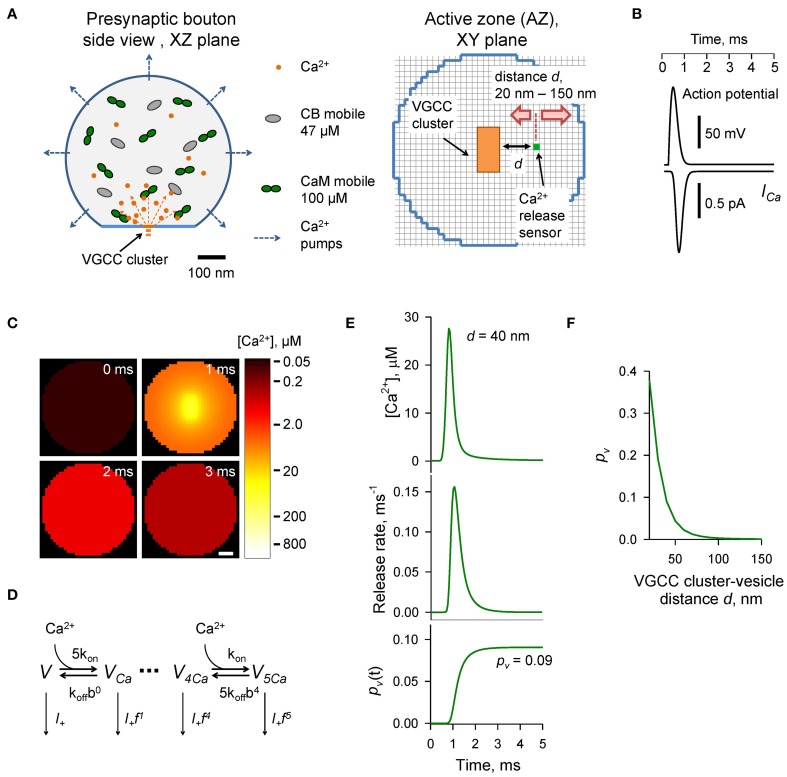
**Modeling of AP-evoked synaptic vesicle exocytosis in a small presynaptic bouton**. **(A)** Presynaptic bouton geometry. Left, side view of a bouton modeled as a truncated sphere of *R_bout_* = 0.3 μm. Right, the AZ plane containing the VGCC cluster modeled as a 40 × 80 nm rectangle (orange); red arrows depict the range of tested coupling distances *d* (20–150 nm) between the VGCC cluster and the vesicular Ca^2+^ release sensor (green dot). Grid 10 nm. **(B)** AP waveform (top trace) and corresponding average Ca^2+^ current *I_Ca_* (bottom trace) through 7 P/Q-type, 8 N-type, and 1 R-type VGCCs. **(C)** Snapshots of spatial AP-evoked [Ca^2+^] within a 10 nm thick plane immediately above the AZ, scale bar 50 nm. Logarithmic color coding bar is shown on the right. **(D)** Allosteric model of Ca^2+^ activation of vesicle fusion (Lou et al., 2005) **(E)** Results of simulations for a representative VGCC—Ca^2+^ sensor coupling distance *d* = 40 nm: [Ca^2+^] time course (top trace), corresponding vesicular release rate time course (middle trace), and time dependency of cumulative vesicular release probability *p_v_*(*t*) (bottom trace). Final AP-evoked vesicular release probability *p_v_* is shown in the insert. This was determined as the horizontal asymptote of a cumulative probability after the AP (in practice we defined it as a value of *p_v_*(*t*) at 5 ms) **(F)** Dependency of *p_v_* on distance *d*.

The presynaptic bouton was considered as a truncated sphere (Figure 1A) of radius *R_bout_* = 0.3 μm (described by the equation [*x*^2^ + *y*^2^ + *z*^2^ ≤ 0.09] · [*z* ≤ 0.25], all distances are in μm). The AZ containing VGCCs was modeled as a circle of radius *R_AZ_* = 0.16 μm situated in the center of the truncated plane *z* = 0.25 μm. VGCCs were evenly distributed within a rectangular cluster (40 by 80 nm) which was placed in the center of the AZ. The cluster contained 7 P/Q-type, 8 N-type, and 1 R-type VGCCs (Ermolyuk et al., 2013).

The model assumed Ca^2+^ binding to the three endogenous buffers present in the presynaptic bouton: CaM, CB, and ATP. Ca^2+^ interaction with free CaM was simulated using a two-step cooperative binding model to the N- and the C-lobes of CaM molecule (Faas et al., 2011):

$$N_{T}N_{T}+Ca^{2+}\overset{2\cdot k_{on}^{(T),N}}{\underset{k_{off}^{(T),N}}{⇄}}CaN_{T}N_{R}+Ca^{2+}\overset{k_{on}^{(R),N}}{\underset{2\cdot k_{off}^{(R),N}}{⇄}}CaN_{R}CaN_{R},$$

$$C_{T}C_{T}+Ca^{2+}\overset{2\cdot k_{on}^{(T),C}}{\underset{k_{off}^{(T),C}}{⇄}}CaC_{T}C_{R}+Ca^{2+}\overset{k_{on}^{(R),C}}{\underset{2\cdot k_{off}^{(R),C}}{⇄}}CaC_{R}CaC_{R},$$

*k^(T),N^_on_* = 770 μM^−1^ s^−1^, *k^(T),N^_off_* = 1.6 × 10^5^ s^−1^, *k^(R),N^_on_* = 3.2 × 10^4^ μM^−1^ s^−1^, *k^(R),N^_off_* = 2.2 × 10^4^ s^−1^, *k^(T),C^_on_* = 84 μM^−1^ s^−1^, *k^(T),C^_off_* = 2.6 × 10^3^ s^−1^, *k^(R),C^_on_* = 25 μM^−1^ s^−1^, *k^(R),C^_off_* = 6.5 s^−1^. The total average CaM concentration was [*CaM*]_*tot*_ = 100 μM (Faas et al., 2011). Depending on the type of simulation (as indicated in the text) CaM was considered either as a mobile buffer with diffusion coefficient *D_CaM_* = 20 μm^2^ s^−1^, or as an immobile buffer which was either evenly distributed throughout the bouton volume or bound to the plasma membrane (within a 10 nm single layer adjacent to the bouton membrane in VCell simulations). In the case of CaM associated with neuromodulin we assumed that *k^(R),C^_off_* was increased 50-fold (Gaertner et al., 2004; Hoffman et al., 2014) (*k^(R),C^_off_* = 325 s^−1^).

Each CB molecule contained four independent Ca^2+^ binding sites (two fast and two slow) (Nagerl et al., 2000):

$$\begin{array}{l} CB_{fast}+Ca^{2+}\overset{k_{on}^{CB\_fast}}{\underset{k_{off}^{CB\_fast}}{⇄}}CaCB_{fast},\\ CB_{slow}+Ca^{2+}\overset{k_{on}^{CB\_slow}}{\underset{k_{off}^{CB\_slow}}{⇄}}CaCB_{slow}, \end{array}$$

*k^CB_fast^_on_* = 87 μM^−1^ s^−1^, *k^CB_fast^_off_* = 35.8 s^−1^, *k^CB_slow^_on_* = 11 μM^−1^ s^−1^, *k^CB_slow^_off_* = 2.6 s^−1^. The diffusion coefficient for both Ca^2+^-free and Ca^2+^-bound CB molecules was *D_CB_* = 20 μm^2^ s^−1^ and the total CB concentration was [*CB*]_*tot*_ = 47.5 μM (Muller et al., 2005).

Ca^2+^ binding to ATP was modeled as a second order reaction:

$$ATP+Ca^{2+}\overset{k_{on}^{ATP}}{\underset{k_{off}^{ATP}}{⇄}}CaATP,$$

*k^ATP^_on_* = 500 μM^−1^ s^−1^, *k^ATP^_off_* = 1.0 × 10^5^ s^−1^. The diffusion coefficient of free and Ca^2+^ bound ATP was *D_ATP_* = 220 μm^2^ s^−1^ (Meinrenken et al., 2002). The total ATP concentration was *[ATP]_tot_* = 0.9 mM, corresponding to 58 μM *[ATP]_free_* at resting physiological conditions (assuming 1 mM intracellular [Mg^2+^]) (Faas et al., 2011).

Ca^2+^ extrusion by the bouton surface pumps (excluding the AZ) was approximated by a first-order reaction: *j_extr_* = −*k_extr_* · ([*Ca*^2+^]−[*Ca*^2+^]*_rest_*) (Matveev et al., 2006; Ermolyuk et al., 2013), with *k_extr_* = 125 μm s^−1^ and [*Ca*^2+^]*_rest_* = 50 nM.

AP-evoked Ca^2+^ currents through P/Q-, N-, and R-type VGCCs were modeled in the NEURON simulation environment (Hines and Carnevale, 1997) using a six-state channel gating kinetic model of P/Q-, N-, and R-type VGCCs in hippocampal mossy fiber boutons (Li et al., 2007) as described in detail previously (Ermolyuk et al., 2013). The mean AP-evoked Ca^2+^ current at the VGCC cluster was approximated by averaging 500 independent NEURON simulations of AP-evoked Ca^2+^ currents for each channel sub-type, followed by fitting the sum of average Ca^2+^ currents corresponding to 7 P/Q-type, 8 N-type, and 1 R-type VGCCs with the function *I_Ca_*(*t*) = $\frac{A}{t}$ exp [−*B* · [ln(*t/t*_0_)]^2^], where *A* = 9.2246 × 10^−4^ pA s, *B* = 15.78, *t*_0_ = 8.036 × 10^−4^ s (Figure 1B). We did not consider any possible effects of AP waveform changes during repeated AP stimulations and assumed that the magnitude of Ca^2+^ influx was the same at each AP. Access to the VCell simulations is available upon request.

### Modeling of Ca^2+^-triggered synaptic vesicle fusion

Vesicular release rates were calculated using a previously published six-state allosteric model of Ca^2+^ activation of vesicle fusion in the calyx of Held (Lou et al., 2005) (Figure 1D). The model parameters were: *k_on_* = 1 × 10^8^ M^−1^ s^−1^, *k_off_* = 4 × 10^3^ s^−1^, *b* = 0.5, *f* = 31.3, and *I*_+_ = 2 × 10^−4^ s^−1^. The model was solved using a variable-order stiff multistep method based on the numerical differentiation formulas (function *ode15s* in MATLAB, MathWorks USA) for AP-evoked Ca^2+^ concentration profiles obtained in VCell simulations at each of the 10 × 10 × 10 nm voxels located immediately above the AZ plane (Figure 1C). MATLAB computer code is enclosed (Supplementary MATLAB code).

The time-dependent vesicular release probability at each voxel in the AZ was calculated as *p_v_*(*t*) = 1 − ∑*_i_ V_i_*(*t*), where ∑*_i_ V_i_*(*t*) is the sum of occupancies of all six model states *V_i_* (Figure 1D). The release rate was then calculated as *R_rel_* = *dp_v_*(*t*)/*dt*. In this work we were specifically interested in dissecting the relative effects of CaM and CB on vesicular release and short-term facilitation. Therefore, we did not take into account any changes in the number of release-ready vesicles that occur during paired-pulse stimulation due to vesicle depletion and replenishment. We thus considered that at the onsets of both the first and second APs the vesicular release sensor was in Ca^2+^ unbound state *V*_*t* = 0*ms*_ = *V*_*t* = 20*ms*_ = (1,0,0,0,0,0). To account for sensitivity of AP-evoked release observed in small excitatory hippocampal and neocortical synapses to the slow endogenous buffer EGTA (e.g., Rozov et al., 2001; Ermolyuk et al., 2013), voxels located closer than 20 nm to the VGCC clusters were excluded from the analysis.

## Results

### Experimentally constrained model of AP-evoked synaptic vesicle exocytosis in small central synapses

To compare the effects of CB and CaM Ca^2+^ buffering on AP-evoked vesicular release and short-term synaptic plasticity we used a realistic experimentally constrained three-dimensional model of AP-evoked VGCC-mediated Ca^2+^ influx, Ca^2+^ buffering and diffusion, and Ca^2+^-dependent activation of vesicular release sensors. The modeling framework consisted of two steps: simulation of buffered Ca^2+^ diffusion in the presynaptic bouton using VCell environment, and calculation of vesicular release rates and fusion probabilities *p_v_* using an allosteric model of the Ca^2+^ activation of vesicle fusion developed in the calyx of Held (Lou et al., 2005) (Materials and Methods).

The presynaptic bouton was considered as a truncated sphere (*R_bout_* = 0.3 μm) with the AZ located at the truncated plane (Figure 1A). Immunogold electron microscopy has shown that P/Q-type VGCCs in small excitatory CA3 hippocampal synapses are mainly situated in small oval-shaped clusters with typical dimensions of 50–100 nm, and that the number of such clusters linearly scales with the size of the AZ (Holderith et al., 2012). To simplify our model we assumed that the AZ had only a single VGCC cluster of rectangular shape: 40 × 80 nm (Figure 1A). Indeed, several studies have argued that for a given release-ready vesicle docked at the AZ its AP-evoked release probability *p_v_* is mainly determined by the closest VGCC cluster (Meinrenken et al., 2002; Ermolyuk et al., 2013; Nakamura et al., 2015).

AP-evoked release in small central excitatory synapses is triggered by mixed populations of P/Q-, N-, and R-type VGCCs (Wu and Saggau, 1994; Reid et al., 1998; Li et al., 2007; Holderith et al., 2012; Sheng et al., 2012). Based on experimental data for the relative numbers of P/Q-, N-, and R-type VGCCs in small hippocampal boutons (Ermolyuk et al., 2013) and for the average channel density within VGCC clusters (Holderith et al., 2012) we considered that the VGCC cluster contains 7 P/Q-type, 8 N-type, and 1 R-type VGCCs. In this simplified model we did not take into account the stochastic behavior of individual VGCCs during an AP and assumed that all channels are evenly distributed within the VGCC cluster. Thus, total AP-evoked Ca^2+^ influx at the VGCC cluster was approximated as the sum of average Ca^2+^ currents corresponding to 7 P/Q-type, 8 N-type, and 1 R-type VGCCs (Figure 1B and Materials and Methods).

We considered that in addition to ATP the presynaptic bouton contains two major presynaptic Ca^2+^ buffers found in central excitatory synapses: CB [physiological [*CB*]_*tot*_ ~ 47.5 μM, total concentration of Ca^2+^ binding sites 190 μM; (Berggard et al., 2002; Jackson and Redman, 2003; Muller et al., 2005; Scott and Rusakov, 2006)] and CaM (physiological [*CaM*]_*tot*_ ~ 100 μM, total concentration of Ca^2+^ binding sites 400 μM; Faas et al., 2011). In the first set of simulations we assumed that CaM molecules are mobile and have the same coefficient of diffusion in Ca^2+^-free and Ca^2+^-bound states equal to that of CB (*D_CaM_* = *D_CB_* = 20 μm^2^ s^−1^).

To calculate the AP-evoked synaptic vesicle release probability *p_v_* as a function of distance between the VGCC cluster and the vesicular release sensor (coupling distance *d*, Figure 1A) we extracted from the three-dimensional VCell model Ca^2+^ dynamics at the AZ (Figure 1C) and then calculated *p_v_* at different *d* using the allosteric model of Ca^2+^-triggered synaptic vesicle fusion (Figure 1D). Consistent with experimental data (Murthy et al., 2001; Ariel and Ryan, 2010; Ermolyuk et al., 2012) the model predicted that physiologically relevant *p_v_*-values (0.05–0.15 range) should correspond to an average coupling distance *d* within a 30–50 nm range (Figures 1E,F).

### Dominant effect of CaM Ca^2+^ buffering on AP-evoked vesicular release

**Figure 2:**
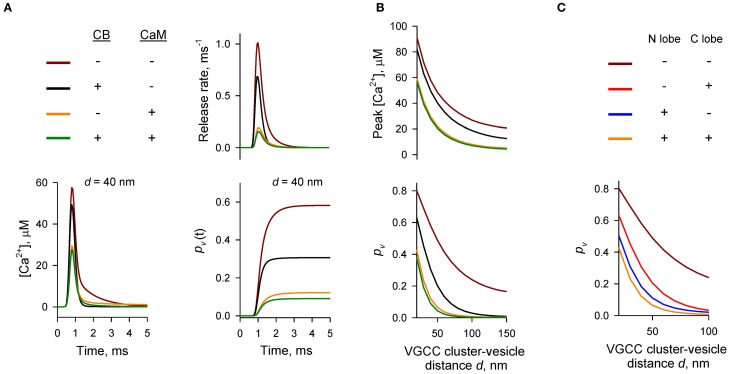
**Dominant effect of CaM on inhibition of AP-evoked vesicular release probability *p_v_***. **(A,B)** Simulation results for different presynaptic Ca^2+^ buffer mixtures, color codes are shown on the top left in **(A)**. **(A)** Time courses for [Ca^2+^] (bottom left), vesicular release rate (top right), and cumulative vesicular release probability *p_v_*(*t*) (bottom right) for a representative VGCC—Ca^2+^ sensor coupling distance *d* = 40 nm. **(B)** Dependencies of peak [Ca^2+^] at release sensor (top) and *p_v_* (bottom) on distance *d*. **(C)** Relative contributions of the N- and C-lobes of CaM to inhibition of *p_v_* at different distances *d*. CB was absent in this set of simulations.

To compare the relative contributions of CB and CaM to buffering of AP-evoked [Ca^2+^] transients at the AZ (and, as a consequence, to inhibition of vesicular release) we performed simulations using different combinations of CB and CaM either absent or present at physiological concentrations (Figures 2A,B). The model predicted that each buffer on its own efficiently inhibited AP-evoked AZ [Ca^2+^] transients and *p_v_*. At a typical coupling distance *d* = 40 nm CB caused ~ 50% reduction of *p_v_* (from 0.58 to 0.31) relative to control simulations without CB and CaM. CaM had even stronger inhibitory effect: ~80% reduction of *p_v_* at *d* = 40 nm (from 0.58 to 0.12). Consistent with the steep power relationship between vesicular release rate and [Ca^2+^] at the release sensors (Mintz et al., 1995; Lou et al., 2005) Ca^2+^ buffering by CB and CaM caused a non-additive supralinear reduction of *p_v_*. Notably, addition of CB on top of CaM caused only a minor further decrease of *p_v_* (e.g., from 80 to 85% at *d* = 40 nm).

We next compared the relative contributions of the fast Ca^2+^ binding to the CaM N-lobe (limiting rate constant *k^(T),N^_on_* = 770 μM^−1^ s^−1^) and the slower Ca^2+^ binding to the CaM C-lobe (limiting rate constant *k^(T),C^_on_* = 84 μM^−1^ s^−1^) to inhibition of *p_v_*. Consistent with its ~ ten-fold higher Ca^2+^ binding rate the N-lobe had a dominant effect in reducing AP-evoked [Ca^2+^] transients at the AZ and *p_v_* (Figure 2C). The C-lobe on its own produced an inhibitory effect similar to that of CB.

Thus, our simulations show that fast synchronous AP-evoked vesicular release at synapses that contain both CB and CaM is mainly inhibited by fast Ca^2+^ binding to the N-lobe of CaM and that the CaM C-lobe and CB play only secondary roles.

### Effect of mobile CaM on paired-pulse facilitation

At certain types of central synapses CB has been shown to contribute to short-term facilitation of AP-evoked vesicular release through Ca^2+^ buffer saturation (e.g., Blatow et al., 2003; Jackson and Redman, 2003). Given the predicted dominant effect of CaM on AP-evoked release we asked how Ca^2+^ buffering by CaM affects short-term synaptic plasticity in presynaptic boutons that contain both CB and CaM. Facilitation through buffer saturation strongly depends on the mobility of the endogenous Ca^2+^ buffers (e.g., Matveev et al., 2004). CaM binds to multiple soluble and membrane-bound proteins (Xia and Storm, 2005; Villarroel et al., 2014). However, the precise distribution of presynaptic CaM molecules between the mobile and immobile states is not known. Therefore, we explored several limiting cases with respect to the diffusional properties and spatial distribution of presynaptic CaM.

**Figure 3:**
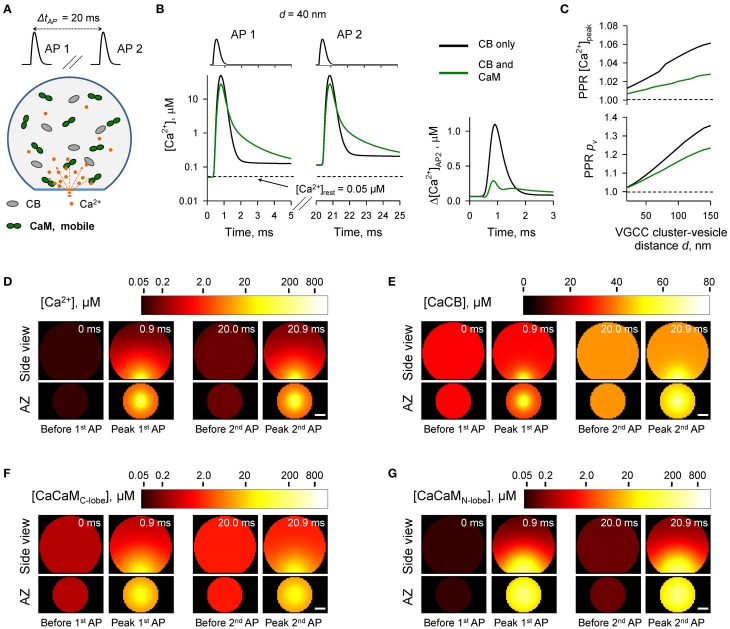
**Effect of mobile CaM on short-term synaptic plasticity**. **(A)** Schematic representation of the paired-pulse simulation experiment (Δ*t_AP_* = 20 ms) with mobile CaM, see text for details. **(B)** Comparison of [Ca^2+^] time courses at the vesicular release Ca^2+^ sensor for a representative coupling distance *d* = 40 nm during paired-pulse stimulation (AP 1 and AP 2) with and without mobile CaM in the presynaptic bouton. Left, [Ca^2+^] transients; right, net increase of [Ca^2+^] at the second AP (Δ[*Ca*^2+^]_*AP*2_ = [*Ca*^2+^]_*AP*2_ − [*Ca*^2+^]_*AP*1_). **(C)** Dependencies of *PPR*[*Ca*^2+^]_*peak*_ = [*Ca*^2+^]^*AP*2^*_peak_*/[*Ca*^2+^]^*AP*1^*_peak_* and *PPR_p_v__* = *p_v_*^*AP*2^/*p_v_*^*AP*1^ on the coupling distance *d*. *p_v_*^*AP*1^ and *p_v_*^*AP*2^ were determined as the horizontal asymptotes of *p_v_*^*AP*1^(*t*) and *p_v_*^*AP*2^(*t*) after the first and the second APs respectively. **(D–G)** Snapshots of spatial distribution of Ca^2+^ (*D*, [*Ca*^2+^]), Ca^2+^ bound to CB (E, [CaCB] = [CaCB_fast_] + [CaCB_slow_]), and Ca^2+^ bound to the C-lobe (F, [CaCaM_C-lobe_] = [CaC_T_C_R_] + 2 · [CaC_R_CaC_R_]) and the N-lobe (G, [CaCaM_N-lobe_] = [CaN_T_N_R_] + 2 · [CaN_R_CaN_R_]) of CaM during paired-pulse stimulation. Side view, XZ plane through the center of the bouton (as in **A**); AZ, 10 nm thick plane immediately above the AZ. Scale bar 100 nm.

We first considered the case of mobile CaM (Figure 3, see also Figures 1, 2). We modeled Ca^2+^ dynamics and vesicular release during 50 Hz paired-pulse AP stimulation (inter-spike interval Δ*t_AP_* = 20 ms) and calculated the dependencies of paired-pulse ratios (PPRs) on the coupling distance *d* both for peak [Ca^2+^] (*PPR* [*Ca*^2+^]*_peak_* = [*Ca*^2+^]^*AP*2^*_peak_*/[*Ca*^2+^]^*AP*1^*_peak_*) and for the vesicular release probability (*PPR_p_v__* = *p*^*AP*2^_*v*_/*p*^*AP*1^_*v*_) (Figures 3A–C). It should be noted that because we were specifically interested in the effects of CaM and CB on shaping the vesicular release, when calculating *PPR_p_v__* we did not consider any changes in the number of release-ready vesicles that may occur as a result of vesicle depletion and replenishment during repetitive stimulation (Materials and Methods).

In comparison to the control simulations where only CB was present, inclusion of mobile CaM led to a noticeable decrease of both *PPR* [*Ca*^2+^]*_peak_* and *PPR_p_v__* (Figure 3C). CB has a relatively high affinity to Ca^2+^ (*K^eff^_D CB_* = 0.31 μM, Supplementary Figure 1) and binds Ca^2+^ ions that enter the bouton during the first AP both within the transient Ca^2+^-nano/microdomain (local [Ca^2+^] up to 10–100 μM within 20–150 nm from the VGCC cluster) and in the rest of the bouton volume (global [Ca^2+^] ~ 1.0–1.5 μM) (Figures 3D,E). Thus, at the onset of the second AP the concentration of free CB binding sites was noticeably reduced in comparison to the first AP (by ~ 10%, from 163.0 to 148.5 μM, Supplementary Figure 2). In contrast both the C- and the N-lobes of CaM have low Ca^2+^ affinities (*K^eff^_D C−lobe_* = 2.84 μM and *K^eff^_D N−lobe_* = 12.0 μM, Supplementary Figure 1) and bind Ca^2+^ ions mainly within the Ca^2+^-nano/microdomain (Figures 3F,G). Therefore, because of the diffusional equilibration at the onset of the second AP over 99% of CaM Ca^2+^ binding sites at the AZ remained in the unbound state (Supplementary Figure 2). Thus, the presence of mobile CaM, which directly competes with CB for Ca^2+^ in the AZ, occludes the short-term facilitation caused by saturation of CB.

### Effect of immobile CaM on paired-pulse facilitation

**Figure 4:**
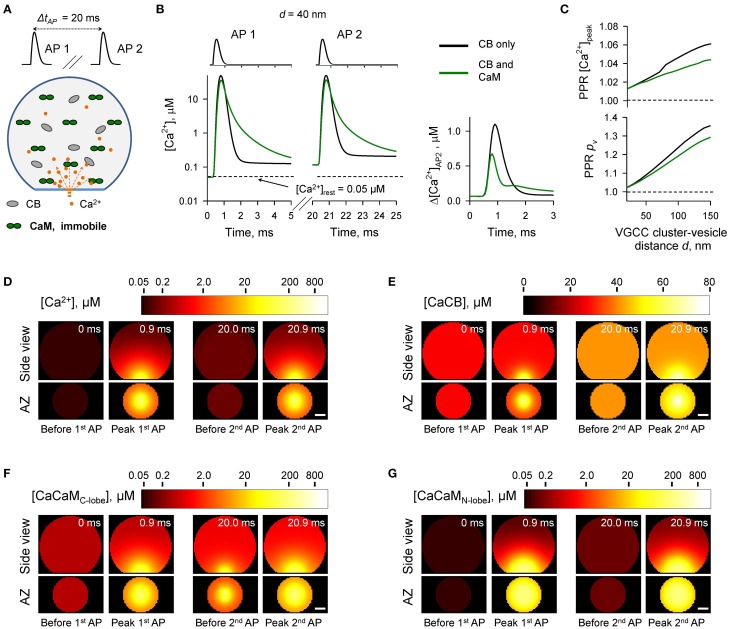
**Effect of immobile CaM on short-term synaptic plasticity**. **(A)** Schematic representation of the paired-pulse simulation experiment (Δ*t_AP_* = 20 ms) with immobile CaM, see text for details. **(B)** Comparison of [Ca^2+^] time courses at the vesicular release Ca^2+^ sensor for a representative coupling distance *d* = 40 nm during paired-pulse stimulation (AP 1 and AP 2) with and without immobile CaM in the presynaptic bouton. Left, [Ca^2+^] transients; right, net increase of [Ca^2+^] at the second AP. **(C)** Dependencies of *PPR*[*Ca*^2+^]*_peak_* and *PPR_p_v__* on the coupling distance *d*. **(D–G)** Snapshots of spatial distribution of Ca^2+^ (*D*), Ca^2+^ bound to CB (**E**), and Ca^2+^ bound to the C-lobe (**F**) and the N-lobe (**G**) of CaM during paired-pulse stimulation. Side view, XZ plane through the center of the bouton (as in **A**); AZ, 10 nm thick plane immediately above the AZ. Scale bar 100 nm.

In the next set of simulations (Figure 4) we considered another limiting case and assumed that all CaM molecules were immobile (e.g., bound to immobile target proteins) and were evenly distributed throughout the bouton volume. The presence of immobile CaM still led to a reduction of paired-pulse facilitation mediated by buffer saturation, although on a smaller scale than in the case of mobile CaM (Figures 4A–C). This was due to the contribution of partial saturation of the immobile CaM C-lobe within the Ca^2+^-nano/microdomain (Figure 4F, snapshot “Before 2^nd^ AP”). Ca^2+^ unbinding from the fully occupied C-lobe occurs on a longer timescale (Ca^2+^ dwell time ~ 150 ms, *k^(R),C^_off_* = 6.5 s^−1^) than the 20 ms inter-spike interval. Therefore, at a typical coupling distance *d* = 40 nm only 80% of Ca^2+^ binding sites on the C-lobe were free at the onset of the second AP (Supplementary Figure 3). In contrast Ca^2+^ unbinding from the N-lobe occurs on a much faster timescale (Ca^2+^ dwell time ~ 0.05 ms, *k^(R),N^_off_* = 2.2 × 10^4^ s^−1^). Therefore, concentrations of the available N-lobe Ca^2+^ binding sites were similar at the onsets of the first and the second APs, which led to occlusion of the paired-pulse facilitation caused by saturation of CB and the C-lobe of CaM. In this set of simulations we used Ca^2+^ binding kinetics determined for free CaM (Faas et al., 2011). However, CaM Ca^2+^ binding properties are affected by binding of CaM to its target proteins. These can either increase (e.g., CaM kinase II) or decrease (e.g., neuromodulin) Ca^2+^ affinity of CaM (Gaertner et al., 2004; Xia and Storm, 2005). Therefore, the effects of the immobile CaM on vesicular release probability *p_v_* and short-term plasticity are expected to be also influenced by the distribution of bound CaM among different target proteins.

### The case of membrane-bound CaM

Many CaM binding partners are located on the presynaptic plasma membrane. In particular, neuromodulin is an abundant presynaptic protein which is found in the brain at similar levels to CaM (Alexander et al., 1988; Xia and Storm, 2005; Kumar et al., 2013). Neuromodulin is a member of the IQ motif family of CaM-binding proteins which also includes neurogranin and PEP-19 (Putkey et al., 2003; Xia and Storm, 2005). CaM binds to the IQ motif via the C-lobe at low [Ca^2+^], and dissociates when Ca^2+^ levels increase (Alexander et al., 1988; Xia and Storm, 2005; Kumar et al., 2013). It was proposed that at resting [*Ca*^2+^]_*rest*_ most of presynaptic CaM is bound to the membrane anchored neuromodulin (Xia and Storm, 2005). Indeed, our model predicts that at [*Ca*^2+^]_*rest*_ = 50 nM, over 99.8% of CaM C-lobes should be in the Ca^2+^-free apo-state which has high affinity of binding to neuromodulin.

**Figure 5:**
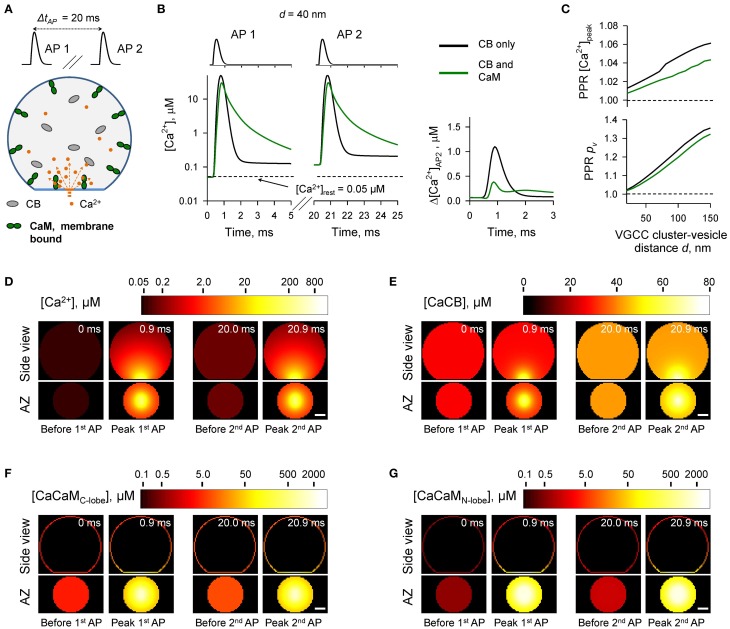
**Effect of membrane-bound CaM on short-term synaptic plasticity**. **(A)** Schematic representation of the paired-pulse simulation experiment (Δ*t_AP_* = 20 ms) with membrane-bound CaM, see text for details. **(B)** Comparison of [Ca^2+^] time courses at the vesicular release Ca^2+^ sensor for a representative coupling distance *d* = 40 nm during paired-pulse stimulation (AP 1 and AP 2) with and without membrane-bound CaM in the presynaptic bouton. Left, [Ca^2+^] transients; right, net increase of [Ca^2+^] at the second AP. **(C)** Dependencies of *PPR*[*Ca*^2+^]*_peak_* and *PPR_p_v__* on the coupling distance *d*. **(D–G)** Snapshots of spatial distribution of Ca^2+^ **(D)**, Ca^2+^ bound to CB **(E)**, and Ca^2+^ bound to the C-lobe **(F)** and the N-lobe **(G)** of CaM during paired-pulse stimulation. Side view, XZ plane through the center of the bouton (as in **A**); AZ, 10 nm thick plane immediately above the AZ. Scale bar 100 nm.

We first considered a limiting case where all CaM molecules were irreversibly bound to neuromodulin molecules located in the bouton plasma membrane. In the VCell simulations we assumed that all CaM molecules were located within a single 10 nm layer adjacent to the plasma membrane (Figure 5). This led to ~ a ten-fold increase of [*CaM*]_*tot*_ near the plasma membrane (1023 μM) in comparison to the case with evenly distributed CaM (100 μM). The detailed Ca^2+^ binding kinetics to CaM associated with neuromodulin remains unknown. However, binding of CaM to the post-synaptically expressed neurogranin (which contains a similar CaM-binding IQ motif) has been shown to decrease Ca^2+^ affinity of the CaM C-lobe because of ~ a fifty-fold acceleration of Ca^2+^ dissociation rate *k^(R),C^_off_* (Gaertner et al., 2004; Hoffman et al., 2014). Therefore, in this set of simulations we also increased *k^(R),C^_off_* 50-fold (from 6.5 to 325 s^−1^).

The simulations revealed that in the case of irreversible binding of CaM to membrane associated neuromodulin, the presence of CaM still partially occludes the short-term facilitation caused by saturation of CB (Figure 5 and Supplementary Figure 4) to the degree similar to that observed in the case of evenly distributed immobile CaM (Figure 4 and Supplementary Figure 3).

### Short-term facilitation through Ca^2+^-induced dislocation of CaM from the plasma membrane

We next considered a more realistic case of dynamic Ca^2+^-dependent interaction between CaM and neuromodulin. Ca^2+^ binding by the C-lobe of CaM reduces its affinity to neuromodulin several fold which leads to dissociation of CaM—neuromodulin complex (Alexander et al., 1988; Kumar et al., 2013; Hoffman et al., 2014). This prompts the hypothesis that Ca^2+^-induced dislocation of CaM molecules from the membrane bound neuromodulin may decrease the Ca^2+^ buffering capacity at the AZ during repetitive AP stimulation, which, in turn, should lead to a use-dependent increase in the vesicular release probability *p_v_*.

**Figure 6:**
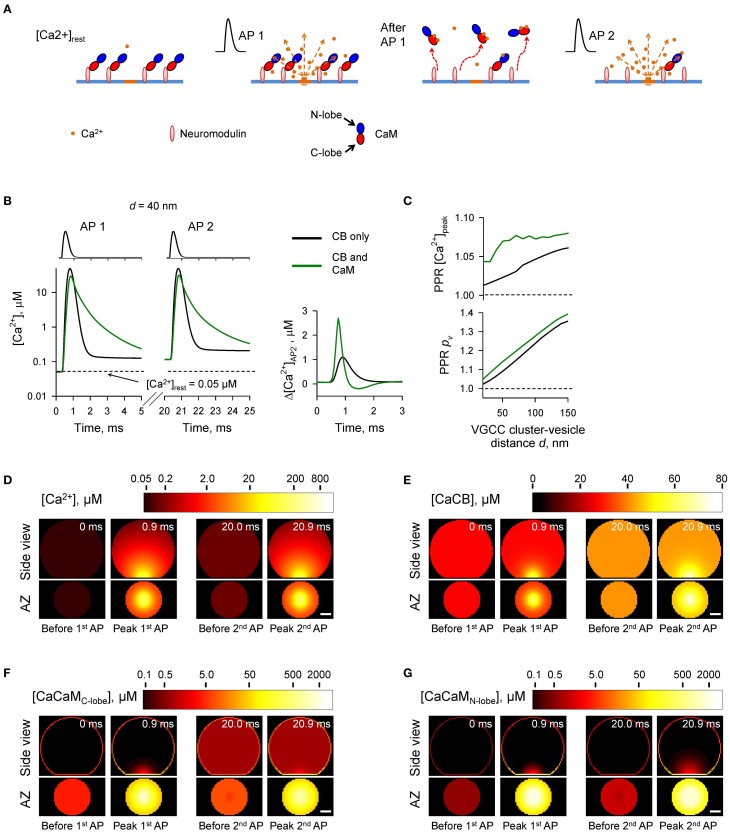
**Ca^2+^-dependent CaM dislocation from the AZ as a mechanism of short-term facilitation**. **(A)** Schematics depicting the model of Ca^2+^-dependent CaM dislocation from the membrane during paired-pulse simulation experiment. We assumed that at resting [*Ca*^2+^]_*rest*_ all CaM molecules were in the Ca^2+^-free apo state and bound via the C-lobes to the membrane-associated neuromodulin molecules. Binding of two Ca^2+^ ions by the C-lobe during the first AP leads to its dissociation from neuromodulin and to reduction of Ca^2+^ buffering at the AZ during the second AP. **(B)** Comparison of [Ca^2+^] time courses at the vesicular release Ca^2+^ sensor for a representative coupling distance *d* = 40 nm during paired-pulse stimulation (AP 1 and AP 2) with and without CaM in the presynaptic bouton. Left, [Ca^2+^] transients; right, net increase of [Ca^2+^] at the second AP. **(C)** Dependencies of *PPR*[*Ca*^2+^]*_peak_* and *PPR_p_v__* on the coupling distance *d*. **(D–G)** Snapshots of spatial distribution of Ca^2+^ **(D)**, Ca^2+^ bound to CB **(E)**, and Ca^2+^ bound to the C-lobe **(F)** and the N-lobe **(G)** of CaM during paired-pulse stimulation. Side view, XZ plane through the center of the bouton (as in **A**); AZ, 10 nm thick plane immediately above the AZ. Scale bar 100 nm.

To test the feasibility of this hypothesis we modeled how Ca^2+^-dependent dislocation of CaM molecules from the plasma membrane to the cytosol affects presynaptic Ca^2+^ dynamics and vesicular release during paired-pulse stimulation (Figure 6). As in Section The Case of Membrane-bound CaM we considered that at the beginning of each simulation ([*Ca*^2+^]_*rest*_ = 50 nM) all CaM molecules were bound to the plasma membrane via the interaction with neuromodulin. We assumed that upon binding of two Ca^2+^ ions by the C-lobe (independently of the Ca^2+^ occupancy of the N-lobe), a CaM molecule can irreversibly dissociate from the plasma membrane and freely diffuse in the cytosol (with *D_CaM_* = 20 μm^2^ s^−1^) (Figure 6A). The dissociation rate of the Ca^2+^ bound C-lobe from neuromodulin (*k^CaM^_off_*) is unknown, but based on thermodynamics principles it is likely to be comparable to the effective Ca^2+^ dissociation rate. Therefore, we assumed that upon Ca^2+^ binding by the C-lobe there is a 50% chance of CaM dissociation from neuromodulin (i.e., *k^CaM^_off_* = 2 · *k^(R),C^_off_* = 650 s^−1^).

Simulations revealed a reduction of [*CaM*]_*tot*_ in the AZ caused by Ca^2+^ influx during the first AP (Figures 6F,G and Supplementary Figure 5). In comparison to the simulations where paired-pulse facilitation was mediated only by the buffer saturation mechanism (Figures 3–5) CaM dislocation led to a noticeably stronger increase in peak [Ca^2+^] and *p_v_* at the second AP (Figures 6B,C). Indeed, in the case of buffer dislocation the decrease of Ca^2+^ buffering at the second AP was not only due to saturation of the relatively slow CB and CaM C-lobe Ca^2+^ binding sites, but also due to a direct reduction in fast Ca^2+^ binding to the N-lobe of CaM, which dominates regulation of fast AP-evoked Ca^2+^-nano/microdomain dynamics and *p_v_* (Figure 2).

**Figure 7:**
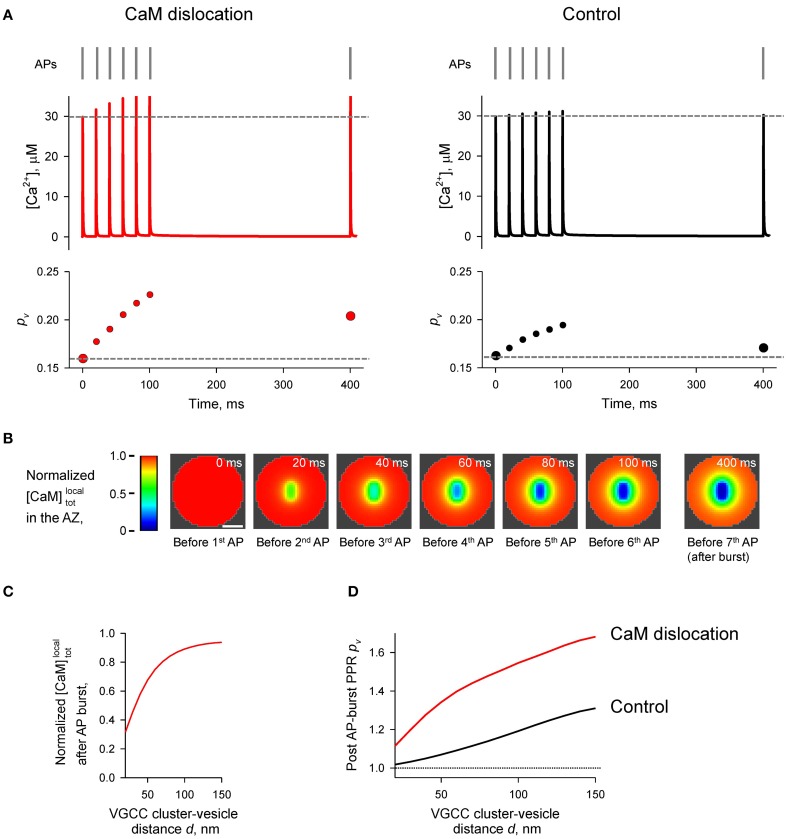
**Effect of CaM dislocation on short-term facilitation during physiological patterns of AP firing**. **(A)** Comparison of Ca^2+^ dynamics and vesicular release probability *p_v_* at a representative coupling distance *d* = 40 nm for the case of Ca^2+^-dependent CaM dislocation (left) and for the control case of irreversible membrane bound CaM (right). Top, physiological AP stimulus pattern; middle, [Ca^2+^] time courses; bottom, *p_v_* plots. **(B)** Snapshots of spatial distribution of normalized local total [*CaM*]*^local^_tot_* (which accounts for all CaM molecules in a given voxel, irrespective of their Ca^2+^ binding state) in the AZ, illustrating progressive dislocation of CaM from the AZ during the burst of APs [the same stimulation pattern as in **(A)**]. Scale bar 100 nm. **(C)** Dependency of normalized [*CaM*]*^local^_tot_* after the burst of APs on the coupling distance *d*. Normalized [*CaM*]*^local^_tot_* in **(B)** and **(C)** were calculated by dividing the spatially dependent [*CaM*]*^local^_tot_* by the initial total [*CaM*]_*tot*_ = 1023 μM. **(D)** Dependences of post-AP burst *PPR_p_v__* on the coupling distance *d* calculated for the single AP at *t* = 400 ms and for the first AP in the burst.

Finally we considered the effect of CaM membrane dislocation on AP-evoked release during physiological firing patterns typical for CA1 hippocampal pyramidal cells. These are characterized by short high-frequency bursts of APs that are interleaved by single APs (O'Keefe and Dostrovsky, 1971; Dobrunz and Stevens, 1999). We modeled AP-evoked presynaptic Ca^2+^ dynamics and vesicular release during a 50 Hz burst of six APs which was followed by a single AP 300 ms after the burst (Figure 7A). The results of our simulations show that cumulative dislocation of CaM from the AZ plasma membrane during the AP burst leads to a prominent and lasting longer facilitation of vesicular release, as evidently from the comparison with the control simulations where all CaM molecules were irreversibly bound to the plasma membrane (Figure 7).

## Discussion

This modeling study investigates the effects of Ca^2+^ buffering by CaM on AP-evoked synaptic vesicle release and short-term synaptic plasticity. The multiple roles of CaM in modulating synaptic transmission, which it exerts via interactions with its target proteins, have been extensively characterized (Xia and Storm, 2005; Pang et al., 2010; Sun et al., 2010; Lipstein et al., 2013; Ben-Johny and Yue, 2014). Hitherto however, the direct effects of Ca^2+^ buffering by CaM on AP-evoked presynaptic Ca^2+^ dynamics and vesicular release have not been systematically investigated.

We used a realistic three-dimensional computational model of AP-evoked presynaptic [Ca^2+^] dynamics and Ca^2+^-triggered vesicular fusion in small excitatory synapses (Ermolyuk et al., 2013). We systematically compared the effects of physiologically relevant concentrations of CaM and CB (the two major Ca^2+^ buffers found in central excitatory synapses) on vesicular release probability and short-term synaptic plasticity. To constrain the model parameters we used recently published detailed kinetics of Ca^2+^ binding to CaM (Faas et al., 2011), which reveal that the N-lobe of CaM binds Ca^2+^ much faster than any other characterized presynaptic Ca^2+^ buffer, whilst the CaM C-lobe binds Ca^2+^ with a rate comparable to that of CB. Consistently with this, our modeling shows that fast Ca^2+^ binding by the N-lobe of CaM plays a dominant role in shaping [Ca^2+^] within the transient AP-evoked Ca^2+^-nano/microdomains and as a consequence in inhibition of vesicular release probability *p_v_*. In contrast, slower Ca^2+^ binding by the CaM C-lobe and by CB plays only a secondary role.

Our simulations also demonstrate that, depending on its mobility and location, CaM may exert opposite effects on short-term facilitation of synaptic responses. First, the fast Ca^2+^ binding/unbinding by the CaM N-lobe generally occludes paired-pulse facilitation of vesicular release caused by partial saturation of CB and the CaM C-lobe (which release Ca^2+^ on a slow time scale). Such an occlusion mechanism, and possible differences in concentration, location and mobility of CaM may explain why Ca^2+^ saturation of CB contributes to short-term facilitation only in certain types of synapses (e.g., Blatow et al., 2003; Muller et al., 2005; Bornschein et al., 2013).

Second, we propose a novel mechanism of short-term facilitation through Ca^2+^-induced dislocation of CaM from the plasma membrane. It is thought that at resting conditions most of the presynaptic CaM is bound to the membrane-associated protein neuromodulin (Alexander et al., 1988; Xia and Storm, 2005). The binding occurs at low [Ca^2+^] via interaction between the apoCaM C-lobe and the IQ-motif of neuromodulin. Upon Ca^2+^ binding by the C-lobe when [Ca^2+^] increases this interaction becomes weaker and CaM dissociates from neuromodulin (Xia and Storm, 2005; Kumar et al., 2013). Thus, we hypothesize that transient increase of [Ca^2+^] within Ca^2+^-nano/microdomains may lead to a dislocation of CaM molecules from the plasma membrane at the AZ into the cytosol.

Indeed, our simulations show that even a single AP would lead to a reduction in [*CaM*]_*tot*_ in the AZ. Such a stimulation-dependent reduction of Ca^2+^ buffering capacity within the AZ results in a noticeable increase in the paired-pulse ratio when compared to the control simulation with irreversible membrane-bound CaM. The effect of Ca^2+^-dependent CaM dislocation was even more prominent during the physiological burst-like AP firing of pyramidal cells.

When modeling the effect of Ca^2+^-dependent CaM dislocation we assumed that the effective concentration of CaM at the membrane was ~1000 μM (to maintain the experimentally estimated [*CaM*]_*tot*_ in the entire bouton at 100 μM). This corresponds to ~25 CaM molecules located at an average sized AZ with an area *S_AZ_* = 0.04 μm^2^ (Schikorski and Stevens, 1997; Holderith et al., 2012). In reality it is likely that the density of CaM molecules bound at the AZ is even higher than that because at [*Ca*^2+^]_*rest*_ apoCaM molecules are also bound to the presynaptic VGCCs via a similar IQ-motif interaction (Ben-Johny and Yue, 2014).

In this work we used a simplified model that did not take into account the mobility of VGCCs in the presynaptic membrane (Schneider et al., 2015) and also assumed irreversible dissociation of CaM from neuromodulin when both binding sites on the CaM C-lobe were occupied by Ca^2+^ ions. Yet, the detailed kinetics of CaM and neuromodulin interaction in the presence and in the absence of Ca^2+^ remains largely unknown. Thus, further experimental and modeling work is required to obtain more realistic models of the complex kinetics of Ca^2+^-dependent interaction of CaM with its binding partners at the AZ. Furthermore, activity-dependent phosphorylation of neuromodulin and other IQ-motif containing proteins prevents their interaction with CaM (Xia and Storm, 2005; Kumar et al., 2013). This should lead to long-lasting changes in the distribution of CaM molecules between the membrane-bound and mobile states, thus regulating Ca^2+^ buffering capacity at the AZ and *p_v_* on a longer timescale. Our theoretical modeling study thus argues that Ca^2+^-dependent CaM dislocation from the plasma membrane could provide a powerful mechanism for dynamic modulation of vesicular release during physiological patterns of activity, and calls for direct experimental testing of this hypothesis.

## Conflict of interest statement

The authors declare that the research was conducted in the absence of any commercial or financial relationships that could be construed as a potential conflict of interest.
