# Supplementary material for: Calmodulin as a major calcium buffer shaping vesicular release and short-term synaptic plasticity: facilitation through buffer dislocation
Source: Front Cell Neurosci. 2015 Jul 1;9:239. doi: 10.3389/fncel.2015.00239 (PMC4486835; doi:10.3389/fncel.2015.00239)
Supplement: Supplementary file 1 [file Presentation1.PDF]

Calmodulin as a major calcium buffer shaping vesicular release and short-term synaptic plasticity: facilitation through buffer dislocation.

Yulia Timofeeva and Kirill E. Volynski

Supplementary Figures

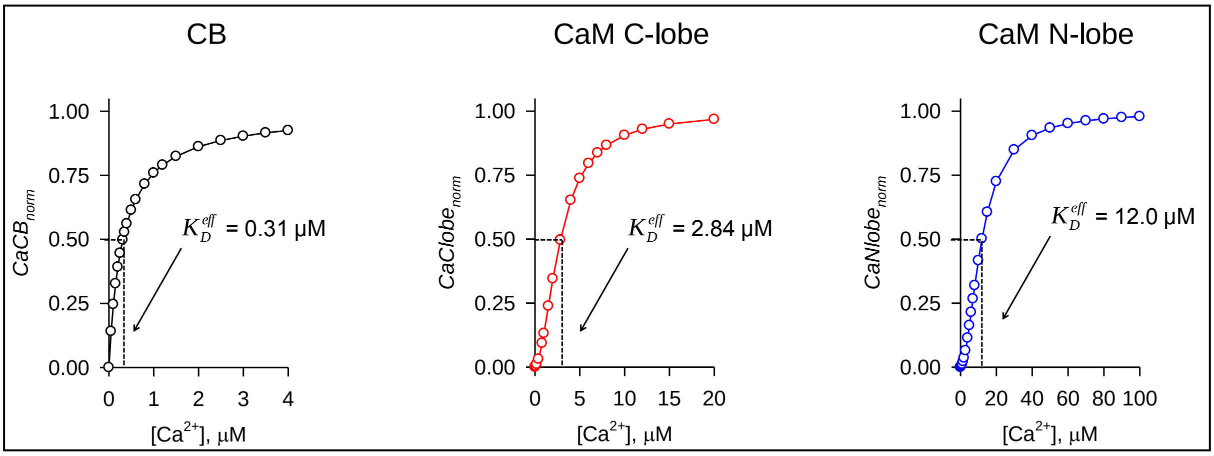

Supplementary Figure 1 │ Ca^{2+} binding by CB, CaM C-lobe and CaM N-lobe at equilibrium.

Normalized Ca^{2+} binding dependencies were calculated as follows:

$$CaCB_{norm} = \frac{[CaCB_{fast}] + [CaCB_{slow}]}{2 \cdot [CB]_{tot}}$$

$$CaClobe_{norm} = \frac{[CaC_T C_R] + 2 \cdot [CaC_R CaC_R]}{2 \cdot [CaM]_{tot}}$$

$$CaNlobe_{norm} = \frac{[CaN_T N_R] + 2 \cdot [CaN_R CaN_R]}{2 \cdot [CaM]_{tot}}$$

Inserts, effective dissociation constants K_D^{eff}, defined as $[Ca^{2+}]$ at which a half of all available Ca^{2+} binding sites are free.

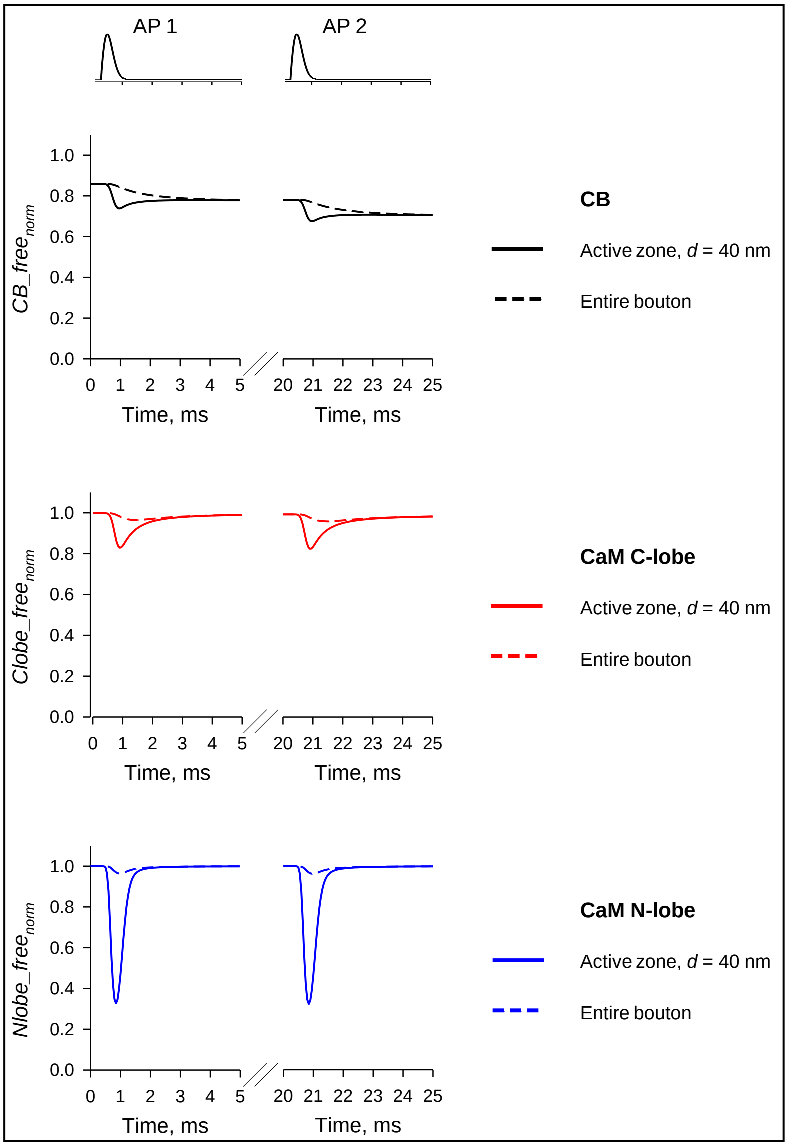

Supplementary Figure 2 │ Case of mobile CaM (related to Figure 3). Comparison of Ca^{2+}-free buffer dynamics for CB, CaM C-lobe and CaM N-lobe at the AZ and in the entire bouton volume.

Dynamics of free Ca^{2+} binding sites (normalized to the total amount of Ca^{2+} binding sites) in the AZ at a typical coupling distance d = 40 nm (solid traces) and integrated through the entire bouton volume (dashed traces). CB (black traces), CaM C-lobe (red traces) and CaM N-lobe (blue traces).

$$CB\_free_{norm} = \frac{[CB_{fast}]+[CB_{slow}]}{2\cdot[CB]_{tot}}$$

$$Clobe\_free_{norm} = \frac{2\cdot[C_T C_T]+[CaC_T C_R]}{2\cdot[CaM]_{tot}}$$

$$Nlobe\_free_{norm} = \frac{2\cdot[N_T N_T]+[CaN_T N_R]}{2\cdot[CaM]_{tot}}$$

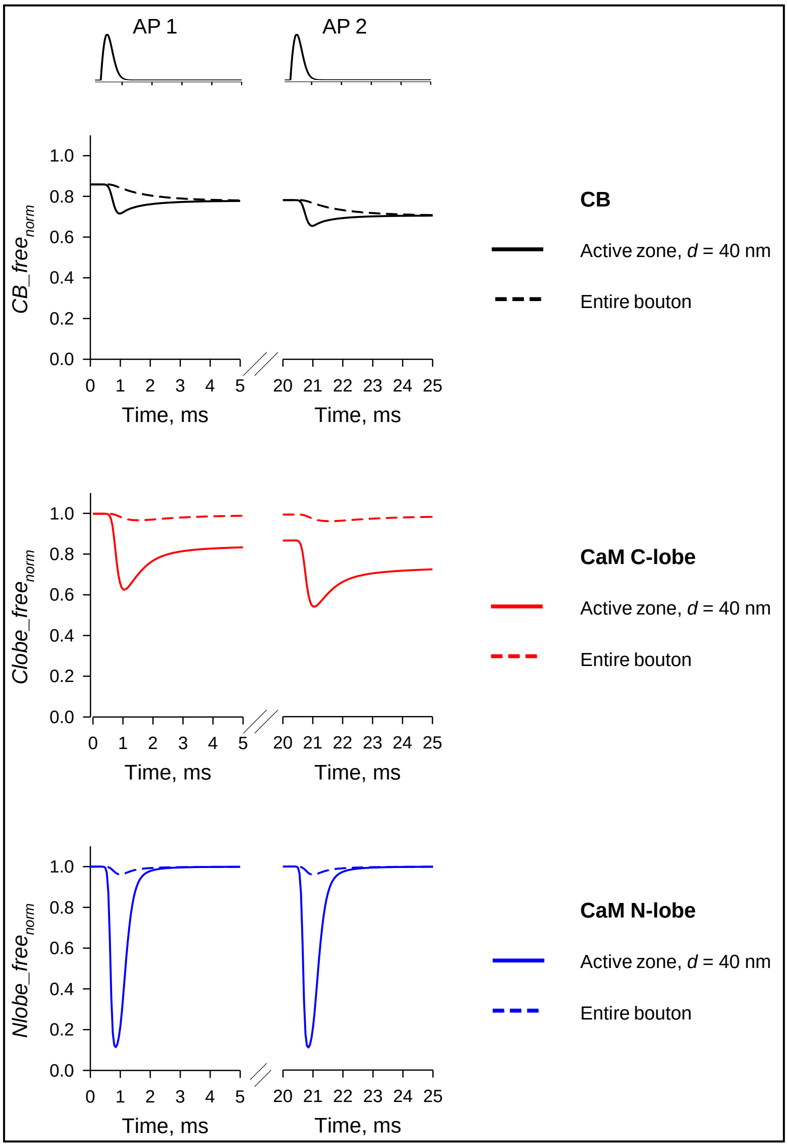

Supplementary Figure 3 │ Case of immobile CaM (related to Figure 4). Comparison of Ca^{2+}-free buffer dynamics for CB, CaM C-lobe and CaM N-lobe at the AZ and in the entire bouton volume.

Dynamics of free Ca^{2+} binding sites (normalized to the total amount of Ca^{2+} binding sites) in the AZ at a typical coupling distance d = 40 nm (solid traces) and integrated through the entire bouton volume (dashed traces). CB (black traces), CaM C-lobe (red traces) and CaM N-lobe (blue traces).

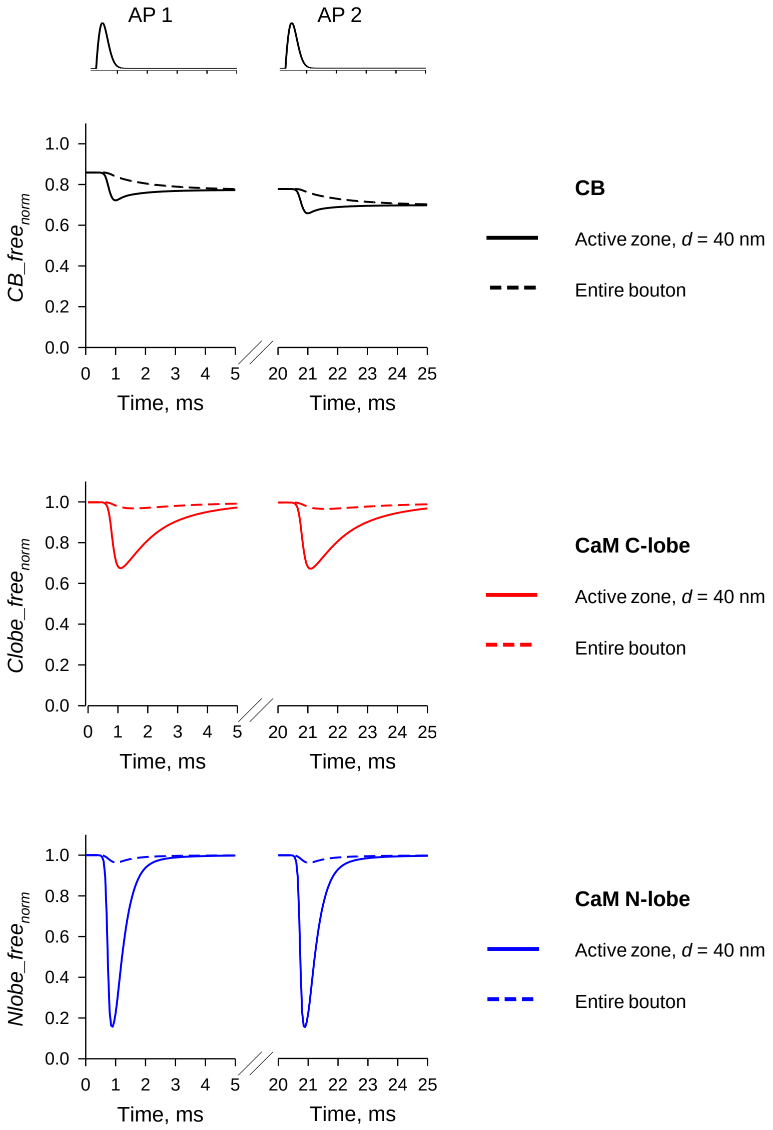

Supplementary Figure 4 │ Case of immobile membrane-bound CaM (related to Figure 5). Comparison of Ca^{2+}-free buffer dynamics for CB, CaM C-lobe and CaM N-lobe at the AZ and in the entire bouton volume.

Dynamics of free Ca^{2+} binding sites (normalized to the total amount of Ca^{2+} binding sites) in the AZ at a typical coupling distance d = 40 nm (solid traces) and integrated through the entire bouton volume (dashed traces). CB (black traces), CaM C-lobe (red traces) and CaM N-lobe (blue traces).

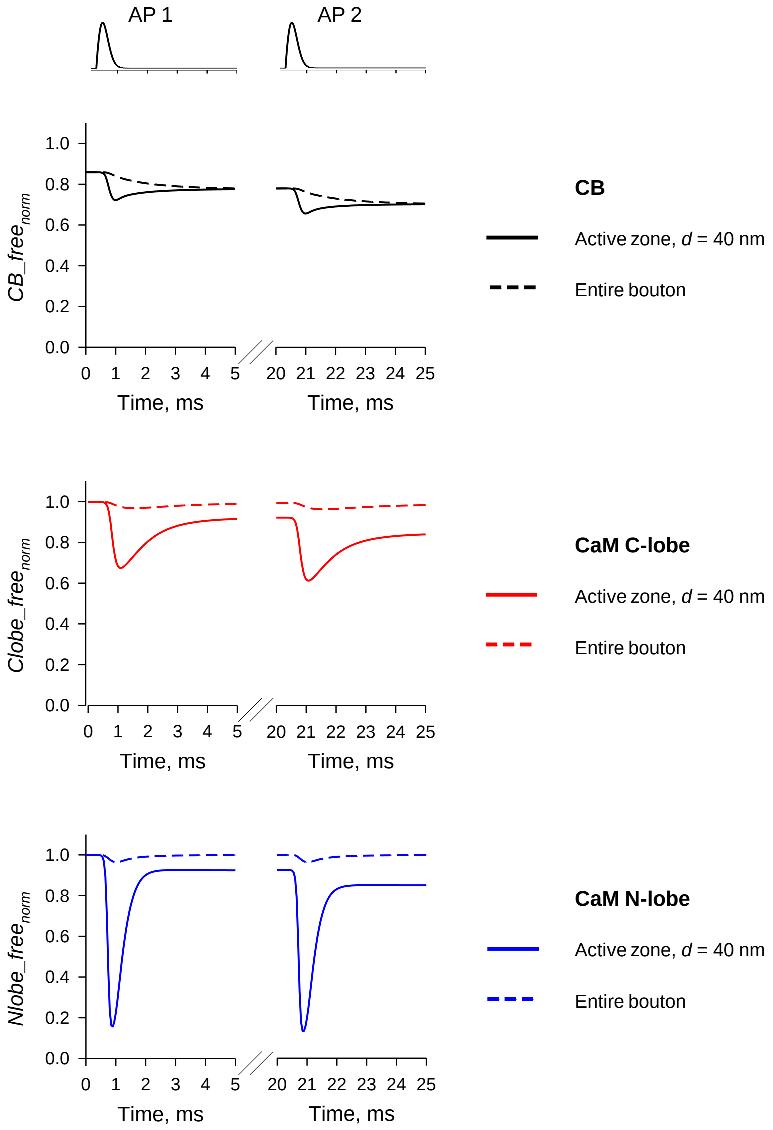

Supplementary Figure 5 │ Case of CaM membrane dislocation (related to Figure 6). Comparison of Ca^{2+}-free buffer dynamics for CB, CaM C-lobe and CaM N-lobe at the AZ and in the entire bouton volume.

Dynamics of free Ca^{2+} binding sites (normalized to the total amount of Ca^{2+} binding sites) in the AZ at a typical coupling distance d = 40 nm (solid traces) and integrated through the entire bouton volume (dashed traces). CB (black traces), CaM C-lobe (red traces) and CaM N-lobe (blue traces).
